# DNA methylation patterns and gene expression from amygdala tissue of mature Brahman cows exposed to prenatal stress

**DOI:** 10.3389/fgene.2022.949309

**Published:** 2022-08-05

**Authors:** Emilie C. Baker, Audrey L. Earnhardt, Kubra Z. Cilkiz, Haley C. Collins, Brittni P. Littlejohn, Rodolfo C. Cardoso, Noushin Ghaffari, Charles R. Long, Penny K. Riggs, Ronald D. Randel, Thomas H. Welsh, David G. Riley

**Affiliations:** ^1^ Department of Animal Science, Texas A&M University, College Station, TX, United States; ^2^ Texas A&M AgriLife Research, College Station, TX, United States; ^3^ Texas A&M AgriLife Research, Overton, TX, United States; ^4^ Department of Computer Science, Prairie View A&M University, Prairie View, TX, United States

**Keywords:** amygdala, Brahman, DNA methylation, gene expression, prenatal stress

## Abstract

Prenatal stress can alter postnatal performance and temperament of cattle. These phenotypic effects may result from changes in gene expression caused by stress-induced epigenetic alterations. Specifically, shifts in gene expression caused by DNA methylation within the brain’s amygdala can result in altered behavior because it regulates fear, stress response and aggression in mammals Thus, the objective of this experiment was to identify DNA methylation and gene expression differences in the amygdala tissue of 5-year-old prenatally stressed (PNS) Brahman cows compared to control cows. Pregnant Brahman cows (*n* = 48) were transported for 2-h periods at 60 ± 5, 80 ± 5, 100 ± 5, 120 ± 5, and 140 ± 5 days of gestation. A non-transported group (*n* = 48) were controls (Control). Amygdala tissue was harvested from 6 PNS and 8 Control cows at 5 years of age. Overall methylation of gene body regions, promoter regions, and cytosine-phosphate-guanine (CpG) islands were compared between the two groups. In total, 202 genes, 134 promoter regions, and 133 CpG islands exhibited differential methylation (FDR ≤ 0.15). Following comparison of gene expression in the amygdala between the PNS and Control cows, 2 differentially expressed genes were identified (FDR ≤ 0.15). The minimal differences observed could be the result of natural changes of DNA methylation and gene expression as an animal ages, or because this degree of transportation stress was not severe enough to cause lasting effects on the offspring. A younger age may be a more appropriate time to assess methylation and gene expression differences produced by prenatal stress.

## 1 Introduction

The amygdala is a cell mass composed of nuclei that are classified into three cell groups located in the temporal cortex of the brain: 1) the basolateral amygdala; 2) cortical like cells; and 3) centromedial cells (Yang and Wang, 2017). The cell groups have neural connections that receive stimuli from areas of the brain including the sensory cortex, the prefrontal cortex, and the hippocampus. It is through those connections the amygdala processes and influences emotions including fear, anxiety, and stress response (Rasia-Filho et al., 2000; Davis and Whalen, 2001). Loss of amygdala function causes emotional based memory loss and aberrant social behavior (Fine and Blair, 2000). In contrast, increased amygdala activity has been linked to various disorders including schizophrenia and bipolar disorder (Lawrie et al., 2003; Kalmar et al., 2009). Increased activation of amygdala neurons can increase vigilance, anxiety, and stress.

The amygdala is a part of the body’s system for detecting stressful and frightening stimuli and then initiating the body’s coping response (LeDoux, 1994). Chronic stress can cause increased anxiety and behavior changes potentially due to hyperexcitability of the amygdala (Rosenkranz et al., 2010). Prenatal stress influences how the amygdala functions by shaping the development and connectivity within it and the tissues it communicates with (Kraszpulski et al., 2006; Scheinost et al., 2016). Shifts in gene expression in the amygdala of prenatally stressed offspring have been observed in mice and sheep (Ward et al., 2000; Petit et al., 2015). The shifts of gene expression in the amygdala may be responsible for the behavioral differences observed in prenatally stressed offspring. Prenatally stressed rhesus monkeys showed altered social behavior including a decrease in play and an increase in clinging to others. When alone the prenatally stressed monkeys showed more inactivity relative to those who did not experience prenatal stress (Clarke et al., 1996). Calves subjected to prenatal transportation stress showed an increase in exit velocity from a restraining chute as well as an increase in temperament score (Littlejohn et al., 2016).

Gene expression shifts in the amygdala of prenatally stressed animals could result from stress-induced DNA methylation alterations. Prenatal stress in Brahman cattle resulted in changes of DNA methylation patterns of leukocytes from 28-day old bull and heifer calves, with differences persisting through 5 years of age (Littlejohn et al., 2018; Baker et al., 2020; Cilkiz et al., 2021). Shifts in DNA methylation patterns have been linked to prenatal stress and changes in temperament of the offspring (Littlejohn et al., 2016; Gartstein and Skinner, 2018; Littlejohn et al., 2018). Methylation of DNA is the addition of a methyl group to the nitrogenous bases in the DNA sequence. In mammals, the addition of the methyl group often occurs to the 5′ carbon of the nitrogenous base cytosine (Razin and Riggs, 1980). Methylation is primarily found within Cytosine-Phosphate-Guanine (CpG) dinucleotides. Methylated cytosines can lead to inhibition of gene expression, while demethylation can promote gene expression. (Tate and Bird, 1993). The methylome changes drastically throughout fetal development and therefore can be influenced by maternal environment. Methylation patterns continue to change postnatally (Salpea et al., 2012). These stress-induced epigenetic modifications can be transgenerational and have the potential to affect many generations in the production system (Feeney et al., 2014; Thompson et al., 2020).

In cattle, the amygdala tissue had the highest percent genome wide DNA methylation relative to other tissues in the limbic system (Cantrell et al., 2019). Considering the amygdala’s important role in behavioral and stress response, modifications to the DNA methylation patterns and gene expression within the amygdala could cause phenotypic differences in the offspring. The long-term phenotypic changes observed in prenatally stressed livestock, including temperament changes, can impact production, animal welfare, and profitable traits (Lay et al., 1997; Cooke, 2014; Serviento et al., 2020). Suckling calves that were exposed to prenatal stress were more temperamental and have a greater serum cortisol concentration than control calves. The early life difference in serum cortisol concentration appears to have been sustained cows selected for harvest at 5 years of age (Control: 29.5 ± 9.8 ng/ml; Prenatally Stressed: 40.34 ± 5.2 ng/ml).

Early life alterations in DNA methylation patterns in humans has measurable effects on behavior and is associated with depression and anxiety (Vonderwalde, 2019). The effects of prenatal stress on methylation and gene expression patterns in the amygdala have been well studied in mice, but less so in livestock species (Kundakovic and Jaric, 2017). Thus, the objective of this study was to investigate whether prenatal stress alters DNA methylation and gene expression in the amygdala of 5-year-old prenatally stressed Brahman cows relative to control cows.

## 2 Methods and materials

All procedures were done in compliance with the Guide for the Care and Use of Agricultural Animals in Research and Teaching (Federation of Animal Science Societies, 2020), and its earlier versions, and approved by the Texas A&M AgriLife Research Animal Care and Use Committee.

### 2.1 Animal procedures

Details of the experimental design and animal handling were described in Littlejohn et al. (2016), Littlejohn et al. (2018), and Cilkiz et al. (2021). Briefly, 96 cows were determined pregnant by rectal palpation 45 days after the breeding date. Cows were then assigned randomly to groups with respect to age, parity, and temperament assessment. The treatment group (*n* = 48) was transported for a duration of 2 h on 60 ± 5, 80 ± 5, 100 ± 5, 120 ± 5, and 140 ± 5 days of gestation (Price et al., 2015). The physiological and metabolic variables measured in the PNS cows were: vaginal temperature (recorded by use of an indwelling vaginal temperature monitoring device), the percentage of weight lost (shrink), and serum concentrations of cortisol and glucose. The dams of the cows used in the present study experienced significantly increased vaginal temperature, shrink, and serum concentrations of cortisol and glucose in response to the transportation events. The findings of Price et al. (2015) reaffirmed our prior reports that transportation constitutes a stressor for pregnant cattle and thereby could influence post-natal development and physiology (Lay et al., 1997; Chen et al., 2015). A group of non-transported cows (*n* = 48) was maintained as a control. Both groups were managed together under the same nutrient and environmental conditions at the Texas A&M AgriLife Research and Extension Center at Overton.

Twenty bull calves and 21 heifer calves were born from the transported cows (PNS), and 26 bull and 18 heifer calves were born to cows that had not been transported (Control). The 39 heifer calves entered a development regimen typical of cows in the herd, which included exposure to bulls for mating at 1 year of age and annually thereafter. Of those females remaining when the group was 5 years old, 8 Control and 6 PNS nonpregnant cows were slaughtered and the whole amygdala from each was harvested and stored at −80°C.

### 2.2 RNA and DNA extraction

Frozen amygdala tissue samples were submitted to the Texas A&M Institute for Genome Sciences and Society (TIGSS) Experimental Genomics Core Laboratory for RNA sequence analysis. The TRIzol Plus RNA Purification Kit (Thermo Scientific, Waltham, MA) was utilized to extract purified RNA from each amygdala sample (approximately 20 mg per sample). Quantification of purified RNA was performed with the Qubit RNA Fluorometric Assay Kit (Thermo Scientific) and the quality was assessed using the RNA ScreenTape Assay (Agilent Technologies, Santa Clara, CA, United States). The RNA was prepared and sequenced with the HS protocol of the Illumina TruSeq Stranded mRNA library preparation kit and mRNA isolated with globin and ribosomal RNA depletion. Paired-end sequencing by the NovaSeq 6000 Sequencing System Illumina Inc.) produced raw RNA FASTQ files as the final output.

Approximately 20 mg of each amygdala tissue sample were digested to extract DNA for methylation analysis. First, 150 μl of sodium chloride-Tris-EDTA buffer, 25 μl of Proteinase K (20 mg/ml) and 25 μl 20% sodium dodecyl sulfate were added to the microcentrifuge tube containing the tissue and gently mixed. Samples were then incubated in a 56°C water bath for 2 h, then 20 μl of RNAse A (10 mg/ml) were added to the sample tubes and the mixture was incubated at 37°C for 30 min. Purified DNA was isolated from the digested amygdala tissue using the protocol for the GeneJET Genomic DNA Purification Kit (Thermo Scientific). Once purified, DNA was quantified with a NanoDrop Spectrophotometer (NanoDrop Technologies, Rockland, DE) and stored at −80°C until further analysis.

### 2.3 DNA methylation library preparation and sequence alignments

Isolated DNA was submitted to Zymo Research (Irvine, CA) for reduced representation bisulfite sequencing methylation analysis. Input DNA was digested with 60 units of TaqαI followed by 30 units of MspI, and then purified with DNA Clean & ConcentratorTM-5. Purified DNA was then ligated to adapters containing 5′-methyl-cytosine. Adapter-ligated fragments of 150–250 bp and 250 to 350 bp were recovered using the ZymocleanTM Gel DNA Recovery Kit. Fragments were then bisulfite-treated using the EZ DNA Methylation-LightningTM Kit followed by preparative-scale PCR and purification.

Standard Illumina base calling was used to identify sequence reads from bisulfite-treated libraries and the raw FASTQ files were trimmed with the TrimGalore 0.6.4 software based upon adapter content and quality. The trimmed sequences were then aligned to the *Bos taurus* genome (ARS-UCD1.2) (Rosen et al., 2020) using Bismark 0.19.0 (Babrahman Bioinformatics, Cambridge, United Kingdom). Methylated and unmethylated read totals at each CpG site were quantified from the aligned binary alignment map (BAM) files using MethylDackel 0.5.0 (Zymo Research).

### 2.4 DNA methylation analysis

#### 2.4.1 Feature specific

Overall methylation of defined features was compared between the PNS and Control groups. The features analyzed included gene bodies, promoter regions (defined as 1,000 bp upstream to 500 bp downstream of the transcription start site), and CpG islands. These features are CpG rich areas of the genome that are vital to epigenetic regulation (Papin et al., 2021). Binary alignment map files that were produced by Zymo Research were read into the SeqMonk program (Babrahman Bioinformatics, Cambridge, United Kingdom). Each feature type was defined, and a bisulfite feature methylation pipeline (SeqMonk) was applied with the requirement of the sites within the feature to have at least 5x coverage, a threshold utilized in other livestock methylation studies (Livernois et al., 2021). Reduced representation bisulfite sequencing can provide accurate analysis at lower coverage, allowing for more biological replicates (Ziller at al., 2015; Crary-Dooley et al., 2017). The pipeline calculates a percentage methylation for each cytosine within the feature and averages these to give an overall value. After the quantification pipeline was applied, a logistic regression was fit, and chi square tests for each feature was performed with the contrast of Control minus PNS. Because this experiment was a very early investigation of methylation in this tissue and species, the false discovery rate (Benjamini and Hochberg, 1995) was controlled at 0.15.

#### 2.4.2 Genome wide methylation

Individual CpG sites across the genome, that is, without regard to predefined features, were also tested. Using the information provided by the methylation call tables the total coverage count, percent methylation, methylated counts, and unmethylated counts were calculated. Sites were filtered in edgeR (Robinson et al., 2010) by requiring 5x coverage at the site in all 14 samples as well as removing sites that were always methylated or unmethylated. A negative binomial generalized linear model was fit to the methylation counts for each site, and a likelihood ratio test was performed at each site using the contrast of Control minus PNS. The false discovery rate (Benjamini and Hochberg, 1995) was controlled at 0.15. Locations in the genome of the significant sites were identified using Ensembl BioMart tool, Ensembl Release 104 (Howe et al., 2020). Multi-Dimensional Scaling (MDS) analysis and plotting were conducted utilizing the M values. The M values are the base 2 logit transformation of the proportion of methylated to unmethylated signal at each locus.

### 2.5 RNA sequence analysis and differential gene expression

Raw RNA FASTQ files were subjected to a 3-step pipeline to generate gene counts. The Trim Galore program (Babrahman Bioinformatics) was used to remove any remaining adapter content. The Spliced Transcripts Alignment to a Reference (STAR) (Dobin et al., 2012) program was used to first create an index file using the ARS-UCD1.2 genome assembly. The trimmed reads were then aligned to the index using the default STAR parameters which had been optimized for alignment of mammalian genomes (Dobin et al., 2012). The BAM files produced by STAR were subjected to procedures of the HTSeq program (Anders et al., 2014) to generate gene counts for each sample.

Differential gene expression analysis was performed in edgeR using a matrix consisting of gene counts from each sample. Genes with no counts were filtered and the remaining counts were normalized using the trimmed mean of M-values method. Tagwise dispersion was calculated, and a negative binomial generalized log-linear model was fit to the read counts for each gene. Finally, a likelihood ratio test corresponding to each gene was calculated with a contrast of Control minus PNS. The false discovery rate was controlled at 0.15 (Benjamini and Hochberg, 1995). Multi-dimensional scaling analysis and plotting were calculated utilizing the normalized read counts.

### 2.6 Cell processes and pathway identification

Further analysis of the significant features and differentially expressed genes was conducted with the PANTHER Classification System 16.0 (Thomas et al., 2003) to identify cellular processes and biological pathways corresponding to identified genes.

## 3 Results and Discussion

### 3.1 DNA methylation

#### 3.1.1 Feature specific-bodies

**FIGURE 1:**
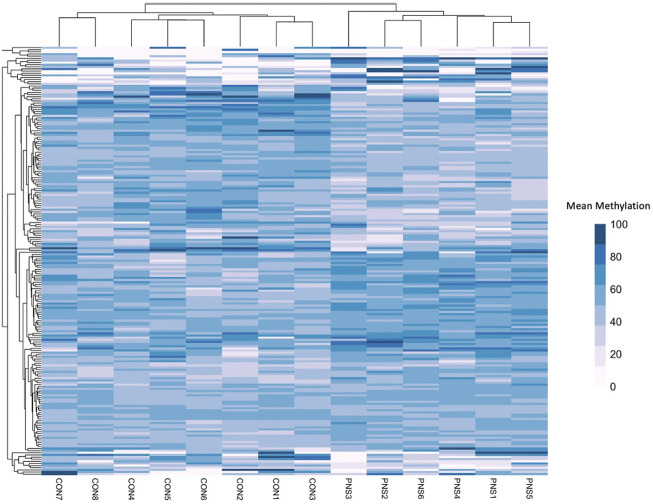
Heatmap showing the mean methylation of the differentially methylated (*n* = 202) genes in the prenatally Stressed (PNS) cows and the Control (CON).

Gene bodies of 26,900 genes were assessed for methylation status. Of those, 202 were differentially methylated between the PNS and Control (FDR ≤ 0.15), with 104 having increased methylation in the PNS group and 98 having decreased methylation (Supplementary Table S1). The top 10 differentially methylated genes in amygdala tissue of prenatally stressed mature Brahman cows relative to Control cows are presented in Table 1. A heatmap of the mean methylation levels of the 202 differentially methylated genes in each sample is presented in Figure 1. Gene body methylation can lead to a decrease in gene expression which can then impact cellular processes (Klose and Bird, 2006). Through use of the PANTHER Classification System, numerous cell processes and biological pathways, including response to stimulus, growth, and metabolic processes, were associated with the differentially methylated genes (Supplementary Table S2). *Dual specificity phosphatase 26* (DUSP26) is active in the oxidative stress response biological pathway, which, in the amygdala contributes to pain response and pain related behavior (Sagalajev et al., 2018). Another highlighted pathway is the ubiquitin proteasome pathway, which is involved in the formation of fear memory within the amygdala (Jarome et al., 2011). Deviations in methylation patterns of genes involved in these pathways could result in altered response to fear and pain in animals.

**TABLE 1:** Top 10 differentially methylated genes in amygdala tissue of prenatally stressed mature to Control cows.

Gene name	Chr	FDR	Difference^a^
*Eukaryotic translation initiation factor 5A2*	1	0.044	15.86
*Homeobox D1*	2	0.061	11.45
*Centrosomal protein 41*	4	0.044	25.61
*Salvador family WW domain containing protein 1*	10	0.003	−17.05
*Brain expressed associated with NEDD4 1*	18	0.003	15.87
*Translocase of outer mitochondrial membrane 40*	18	0.045	17.81
*5S ribosomal RNA*	21	5.68E-18	−3.34
*Ornithine aminotransferase*	26	0.047	12.57
*5.8S ribosomal RNA*	27	0.003	−1.00
*Mitochondrial ribosomal protein L21*	29	0.044	29.03

a A positive (negative) difference indicates the prenatally stressed cows had decreased (increased) methylation of the gene relative to the Control cows.

#### 3.1.2 Feature specific--promoter regions

A total of 134 gene promoters were identified as differentially methylated (FDR ≤ 0.15) in the amygdala tissue of PNS cows when compared to the Control group. Seventy promoter regions had increased methylation in the PNS group, and 64 had decreased methylation (Supplementary Table S3 **)**. The top 10 (lowest FDR value) differentially methylated promoter regions are presented in Table 2. Methylation shifts in promoter regions of genes impact gene expression mainly by influencing the accessibility of the promoter region to transcription factors (Klose and Bird, 2006). One stress-related gene that had increased methylation in its promoter region was *Brain derived neutrophic factor* (*BDNF*). This gene is critical for neural development and function of the amygdala. Alterations of methylation patterns of *BDNF* have been associated with increased anxiety behavior in rats and psychiatric disorders in humans (Redlich et al., 2020). Increased methylation of *BDNF* was observed in individuals that experienced early life stress (Doherty et al., 2016; Blaze et al., 2017). Because of the relationship between *BDNF*, aberrant behavior, and changes in DNA methylation, the methylation of *BDNF* is considered a potential biomarker for early life stress in mammals (Kundakovic et al., 2015). Changes in methylation of this gene could be responsible for the temperament differences that have been observed in prenatally stressed livestock. The stressed group also had decreased methylation of the promoter region of *synapse 1* (*SYN1*). This gene has a role in synaptic function in the amygdala. Male mice who were exposed to early life stress showed an increase in synapse formation and altered synaptic responses (Guadagno et al., 2020). Shifts in gene expression of *SYN1* because of methylation changes could result in altered brain plasticity in the prenatally stress cows. Rats subjected to early maternal separation exhibited increased methylation of *SYN1* (Park et al., 2014) which is contrary to our results of decreased methylation was reported.

**TABLE 2:** Top 10 differentially methylated promoter^a^ regions of genes in amygdala of prenatally stressed mature Brahman cows relative to Control cows.

Gene name	Chr	FDR	Difference^b^
*Oxysterol binding protein like 8*	5	0.005	9.88
*RNA terminal phosphate cyclase like 1*	8	0.001	−13.90
*Maspardin*	10	0.0002	23.28
*WD repeat domain 34*	11	0.005	10.08
*Crumbs cell polarity complex component 1*	16	0.005	−6.87
*5S ribosomal RNA*	21	0.003	-2.24
*Dual Specificity Phosphatase 26*	27	0.006	13.11
*N-deacetylase and N-sulfotransferase 2*	28	0.002	−14.98
*Annexin A8 like 1*	28	0.003	23.37
*Hepatic and glial cell adhesion molecule*	29	0.002	14.75

a Promoter regions were defined as 1,000 base pairs upstream and 500 base pairs downstream from the transcription start site of the gene.

b A positive (negative) difference indicates the prenatally stressed cows had decreased (increased) methylation of the promoter region relative to control cows.

#### 3.1.3 Feature specific--CpG islands

Islands of CpG are often located in promoters of genes, are typically resistant to DNA methylation, and are rarely found in tissue specific genes (Bird, 1986). Because of this it is hypothesized that these regions are in genes that are regularly used in cell function and do not need to be repressed (Bird, 1986). In total 22,188 CpG islands were tested and 133 (FDR ≤ 0.15) were differentially methylated; 77 had increased methylation in the PNS cows and 56 had decreased methylation (Supplementary Table S4). Table 3 has the locations of the top 10 (lowest FDR values) differentially methylated CpG islands identified. A CpG island located within *BDNF* also had increased methylation in the PNS while a CpG island located within the defined promoter region of *SYN1* had decreased methylation. The decrease in methylation of the CpG island within *SYN1* is consistent with what has been reported in aging mice, where decreased methylation of CpG islands within the promoter region coincides with an increase in expression of this gene (Haberman et al., 2012). A CpG island with decreased methylation was located within *Nuclear receptor corepressor 2* (*NCOR2*), which is involved in amygdala development and anxiety behavior (Jessen et al., 2010). The influence of DNA methylation on gene expression of *NCOR2* is relatively unknown, but the location of a CpG island in the regulatory region of the gene suggests the possibility of epigenetic control.

**TABLE 3:** Top 10 differentially methylated CpG Islands^a^ in amygdala tissue of prenatally stressed mature Brahman cows relative to Control cows.

Chr	Start	End	FDR	Difference^b^
4	94192085	94192593	0.040	26.19
7	106955324	106955725	0.058	−35.11
10	43554822	43555817	0.018	−18.35
16	47324909	47326146	0.058	−16.63
18	34194169	34195605	0.003	15.87
19	49916310	49917178	0.058	20.85
20	71009252	71009654	0.058	30.51
21	33001944	33003266	6.99e-18	−3.34
21	33023989	33026059	0.002	2.36
29	42549665	42551050	0.002	14.03

a Cytosine-Phosphate-Guanine rich locations within the genome.

b A positive (negative) difference indicates the prenatally stressed cows had decreased (increased) methylation of the promoter region relative to control cows.

#### 3.1.4 Genome wide methylation

**FIGURE 2:**
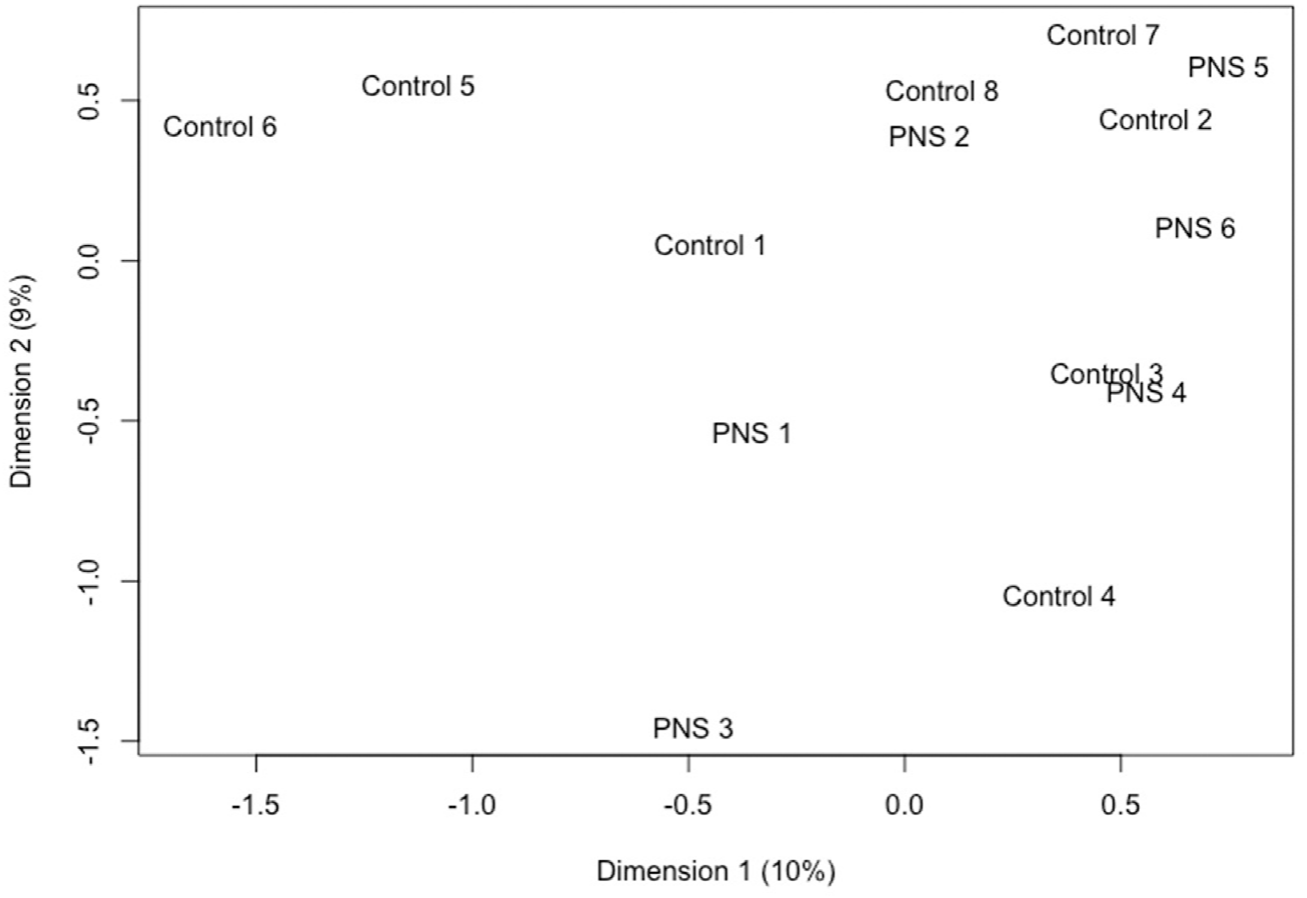
Multidimensional scaling plot utilizing the base 2 logit transformation of the proportion of methylated signal at each locus to plot distances between methylation profiles (M-Values) of amygdala tissue of 5-year-old prenatally stressed (PNS) Brahman cows relative to control cows.

Minimal methylation differences of gene bodies, promoter regions and CpG islands were observed in amygdala tissue between PNS and Control cows at 5 years of age when methylation across the genome was considered in its entirety. Of the genome wide CpG sites, 63,255 sites passed filtering. Only 29 of those sites (Supplementary Table S5) were differentially methylated between the Control and PNS (FDR ≤ 0.15). The significant sites were only 0.046% of the sites tested, indicating that substantial differences in global CpG methylation were not observed between the PNS and Control groups. Visualization of the lack of distinction between treatments is shown in the MDS plot (Figure 2). No distinct grouping of PNS and Control samples occurred and many of the samples from the two treatments were closely positioned. The proximity of the samples to each other in the MDS plot reflects the minimal differences in methylation between groups when evaluated globally. These results differ from analysis of DNA methylation of leukocytes in prenatally stressed Brahman bulls and heifers at 28 days of age which revealed vast differences relative to the Control, some of which were identified in the heifer calves (the cows in this study) and were found in leukocytes 5 years later (Littlejohn et al., 2018; Baker et al., 2020; Cilkiz et al., 2021).

Changes in the epigenetic landscape continue postnatally, with evident differences observed in saliva samples from infants from 6 to 52 weeks of age (Wikenius et al., 2019). In humans, a general trend of demethylation is observed with aging, but some sites that have low methylation at a young age do increase in methylation over time (Wilson et al., 1987; Jones et al., 2015). Differences in methylation caused by the prenatal stress could be present at an early age in cattle but diminish over time. However, severe prenatal stress (i.e., famine and extreme weather) led to lasting DNA methylation changes that were transgenerational (Heijmans et al., 2008; Cao-Lei et al., 2014). The severity of prenatal stress can result in very different outcomes of changes in methylation patterns in the brain (Mychasiuk et al., 2011). Transportation stress during mid to late gestation might not be a severe enough stress to cause enduring epigenetic changes in amygdala tissue in cattle that persist throughout life.

### 3.2 Gene expression

**FIGURE 3:**
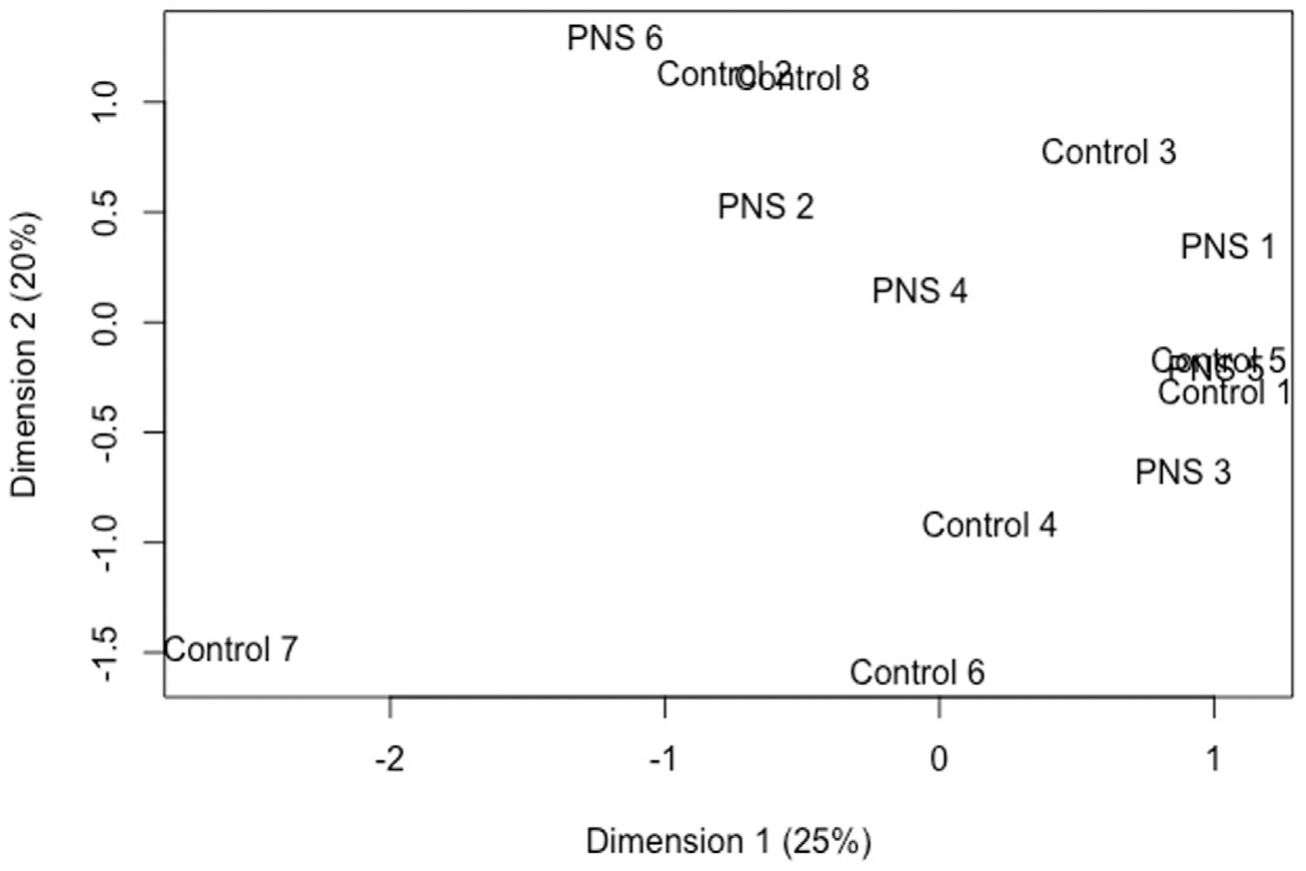
Multidimensional scaling plot utilizing normalized read counts to plot distances between expression profiles of amygdala tissue of 5-year-old prenatally stressed (PNS) Brahman cows relative to Control cows.

From expression analyses, 22,867 genes remained after filtering. Even in the context of a permissive FDR (<0.15), only two genes were differentially expressed in the amygdala of the PNS cows compared to the Controls. The *Solute carrier family 28 member 3* (*SLC28A3*) had decreased expression in the PNS cows relative to the Control, while the *Fc fragment of IgG receptor IIa* (*FCGR2A*) had increased expression. Fc fragment of IgG receptor IIa has an essential role in protecting the body from foreign antigens (Hibbs et al., 1988). Deletion of *FCGR2A* inhibited the invasion of glimoa cells into the brain suggesting the gene product is important for transportation across the blood brain barrier. Members of the solute carrier family are active in the brain, aiding in the transport of hormones, sugars, and amino acids; however, the role of *SLC28A3* in the brain and stress response has not been documented (Hu et al., 2020). The lack of differences is illustrated by the MDS plot (Figure 3) which shows no distinct clustering and some overlap of individual samples from the two groups. There were no methylation differences within the promoter region or gene body of these two differentially expressed genes.

Prenatally stressed Brahman cows had only slight differences in gene expression relative to Controls at 5 years of age. In contrast, in rats, prenatal stress has caused gene expression disturbances in the brain that persisted into adulthood (Fumagalli et al., 2005; Baier et al., 2015). Similar to the DNA methylation results, the timing and severity of a prenatal stressor can dictate the effect on gene expression. Maternal nutrient restriction in cattle has resulted in varying gene expression changes in the offspring depending on timing of restriction during gestation and the tissue analyzed (Mohrhauser et al., 2015; Sanglard et al., 2018). The stress caused by transportation during mid to late gestation may be insufficient to influence gene expression in the offspring. Expression of genes at the proper level is complex, regulated by many different factors, and varies with aging (Berchtold et al., 2008). Corrections may have occurred over time to compensate for aberrant gene expression caused by prenatal stress.

This is this first study to incorporate the effect of prenatal stress on DNA methylation and gene expression in the amygdala of cattle. Overall methylation of important genes and promoter regions were significantly different between the PNS and Control groups. While gene expression analysis resulted in only two significant genes, the two genes are involved in essential biological functions. These novel results provide a foundation for future research on how prenatal stress effects the amygdala in cattle.

## 4 Conclusion

Gene expression and DNA methylation comparison of amygdala tissue from mature Brahman cows that were prenatally stressed relative to non-stressed mature Control cows revealed minimal differences between the groups. A small number of individual CpG sites and a low proportion of genes, promoters and CpG islands were differentially methylated. Two genes were differentially expressed in amygdala tissue when PNS and Control groups were compared. Methylation controls gene expression of many genes; however, no overlap between differentially methylated genes and differentially expressed genes was observed. Since both DNA methylation and gene expression are complex mechanisms that shift and adapt over time, it is feasible that any differences that were caused by the prenatal stress are no longer present at 5 years of age. The timing and severity of the stressor may also be a major influence on the extent of the alterations. Therefore, prenatal transportation stress during mid to late gestation may not be significant enough to cause lasting effects. Increasing the severity of the transportation stress, such as transport for an extended period and over poorer quality of roadways could potentially result in lasting effects. Also, further investigation is needed to determine if there are differences present at younger ages, which could cause expression changes during important postnatal developmental periods. However, much of the current knowledge of the effects of prenatal stress on methylation is from model organisms; thus, the novel information and candidate regions and genes reported are valuable for understanding the effects stress induced epigenetic modifications have on livestock.

## Data Availability

The datasets presented in this study can be found in online repositories. The names of the repository/repositories and accession numbers can be found below: https://www.ncbi.nlm.nih.gov/; SAMN29182872 - SAMN29182885. Data has been uploaded to the NCBI SRA under BioProject PRJNA850510.

## References

[B1] Anders S. Pyl P. T. Huber W. (2014). HTSeq—a Python framework to work with high-throughput sequencing data. Bioinformatics 31 (2), 166–169. 10.1093/bioinformatics/btu638 25260700 PMC4287950

[B2] Baier C. J. Pallarés M. E. Adrover E. Monteleone M. C. Brocco M. A. Barrantes F. J. (2015). Prenatal restraint stress decreases the expression of alpha-7 nicotinic receptor in the brain of adult rat offspring. Stress 18 (4), 435–445. 10.3109/10253890.2015.1022148 25798813

[B3] Baker E. C. Cilkiz K. Z. Riggs P. K. Littlejohn B. P. Long C. R. Welsh T. H. (2020). Effect of prenatal transportation stress on DNA methylation in Brahman heifers. Livest. Sci. 240, 104116. 10.1016/j.livsci.2020.104116

[B4] Benjamini Y. Hochberg Y. (1995). Controlling the false discovery rate: A practical and powerful approach to multiple testing. J. R. Stat. Soc. Ser. B 57 (1), 289–300. 10.1111/j.2517-6161.1995.tb02031.x

[B5] Berchtold N. C. Cribbs D. H. Coleman P. D. Rogers J. Head E. Kim R. (2008). Gene expression changes in the course of normal brain aging are sexually dimorphic. Proc. Natl. Acad. Sci. U. S. A. 105(40), 15605–15610. 10.1073/pnas.0806883105 18832152 PMC2563070

[B6] Bird A. P. (1986). CpG-rich islands and the function of DNA methylation. Nature 321 (6067), 209–213. 10.1038/321209a0 2423876

[B7] Blaze J. Asok A. Borrelli K. Tulbert C. Bollinger J. Ronca A. E. (2017). Intrauterine exposure to maternal stress alters Bdnf IV DNA methylation and telomere length in the brain of adult rat offspring. Int. J. Dev. Neurosci. 62 (1), 56–62. 10.1016/j.ijdevneu.2017.03.007 28330827 PMC5600826

[B8] Cantrell B. Lachance H. Murdoch B. Sjoquist J. Funston R. Weaber R. (2019). Global DNA Methylation in the limbic system of cattle. Epigenomes 3 (2), 8. 10.3390/epigenomes3020008 34968231 PMC8594672

[B9] Cao-Lei L. Massart R. Suderman M. J. Machnes Z. Elgbeili G. Laplante D. P. (2014). DNA methylation signatures triggered by prenatal maternal stress exposure to a natural disaster: Project Ice Storm. PLOS ONE 9 (9), e107653. 10.1371/journal.pone.0107653 25238154 PMC4169571

[B10] Chen Y. Arsenault R. Napper S. Griebel P. (2015). Models and methods to investigate acute stress responses in cattle. Animals 5 (4), 1268–1295. 10.3390/ani5040411 26633525 PMC4693215

[B11] Cilkiz K. Z. Baker E. C. Riggs P. K. Littlejohn B. P. Long C. R. Welsh T. H. (2021). Genome-wide DNA methylation alteration in prenatally stressed Brahman heifer calves with the advancement of age. Epigenetics 16 (5), 519–536. 10.1080/15592294.2020.1805694 32815760 PMC8078662

[B12] Clarke A. S. Soto A. Bergholz T. Schneider M. L. (1996). Maternal gestational stress alters adaptive and social behavior in adolescent rhesus monkey offspring. Infant Behav. Dev. 19 (4), 451–461. 10.1016/S0163-6383(96)90006-5

[B13] Cooke R. F. (2014). Bill E. Kunkle Interdisciplinary Beef Symposium: Temperament and acclimation to human handling influence growth, health, and reproductive responses in *Bos taurus* and *Bos indicus* cattle. J. Anim. Sci. 92 (12), 5325–5333. 10.2527/jas.2014-8017 25023802

[B14] Crary-Dooley F. K. Tam M. E. Dunaway K. W. Hertz-Picciotto I. Schmidt R. J. LaSalle J. M. (2017). A comparison of existing global DNA methylation assays to low-coverage whole-genome bisulfite sequencing for epidemiological studies. Epigenetics 12 (3), 206–214. 10.1080/15592294.2016.1276680 28055307 PMC5406214

[B15] Davis M. Whalen P. J. (2001). The amygdala: vigilance and emotion. Mol. Psychiatry 6 (1), 13–34. 10.1038/sj.mp.4000812 11244481

[B16] Dobin A. Davis C. A. Schlesinger F. Drenkow J. Zaleski C. Jha S. (2012). Star: ultrafast universal RNA-seq aligner. Bioinformatics 29 (1), 15–21. 10.1093/bioinformatics/bts635 23104886 PMC3530905

[B17] Doherty T. S. Forster A. Roth T. L. (2016). Global and gene-specific DNA methylation alterations in the adolescent amygdala and hippocampus in an animal model of caregiver maltreatment. Behav. Brain Res. 298 (Pt A), 55–61. 10.1016/j.bbr.2015.05.028 26027495 PMC4662928

[B18] Federation of Animal Science Societies (FASS) (2020). Guide for the care and use of agricultural animals in research and teaching. 4th ed. Champaign, IL: FASS.

[B19] Feeney A. Nilsson E. Skinner M. K. (2014). Epigenetics and transgenerational inheritance in domesticated farm animals. J. Anim. Sci. Biotechnol. 5 (1), 48. 10.1186/2049-1891-5-48 25810901 PMC4373098

[B20] Fine C. Blair R. J. R. (2000). The cognitive and emotional effects of amygdala damage. Neurocase 6 (6), 435–450. 10.1080/13554790008402715

[B21] Fumagalli F. Bedogni F. Slotkin T. A. Racagni G. Riva M. A. (2005). Prenatal stress elicits regionally selective changes in basal FGF-2 gene expression in adulthood and alters the adult response to acute or chronic stress. Neurobiol. Dis. 20 (3), 731–737. 10.1016/j.nbd.2005.05.005 15967670

[B22] Gartstein M. A. Skinner M. K. (2018). Prenatal influences on temperament development: The role of environmental epigenetics. Dev. Psychopathol. 30 (4), 1269–1303. 10.1017/s0954579417001730 29229018 PMC5997513

[B23] Guadagno A. Verlezza S. Long H. Wong T. P. Walker C.-D. (2020). It is all in the right amygdala: Increased synaptic plasticity and perineuronal nets in male, but not female, juvenile rat pups after exposure to early-life stress. J. Neurosci. 40 (43), 8276–8291. 10.1523/JNEUROSCI.1029-20.2020 32978287 PMC7577595

[B24] Haberman R. P. Quigley C. K. Gallagher M. (2012). Characterization of CpG island DNA methylation of impairment-related genes in a rat model of cognitive aging. Epigenetics 7 (9), 1008–1019. 10.4161/epi.21291 22869088 PMC3515010

[B25] Heijmans B. T. Tobi E. W. Stein A. D. Putter H. Blauw G. J. Susser E. S. (2008). Persistent epigenetic differences associated with prenatal exposure to famine in humans. Proc. Natl. Acad. Sci. U. S. A. 105 (44), 17046–17049. 10.1073/pnas.0806560105 18955703 PMC2579375

[B68] Hibbs M. L. Bonadonna L. Scott B. M. McKenzie I. F. Hogarth P. M. (1988). Molecular cloning of a human immunoglobulin G Fc receptor. PNAS 85 (7), 2240–2244. 10.1073/pnas.85.7.2240 2965389 PMC279966

[B26] Howe K. L. Achuthan P. Allen J. Allen J. Alvarez-Jarreta J. Amode M. R. (2020). Ensembl 2021. Nucleic Acids Res. 49 (D1), D884–D891. 10.1093/nar/gkaa942 PMC7778975 33137190

[B27] Hu C. Tao L. Cao X. Chen L. (2020). The solute carrier transporters and the brain: Physiological and pharmacological implications. Asian J. Pharm. Sci. 15 (2), 131–144. 10.1016/j.ajps.2019.09.002 32373195 PMC7193445

[B28] Jarome T. J. Werner C. T. Kwapis J. L. Helmstetter F. J. (2011). Activity dependent protein degradation is critical for the formation and stability of fear memory in the amygdala. PLoS One 6 (9), e24349. 10.1371/journal.pone.0024349 21961035 PMC3178530

[B29] Jessen H. M. Kolodkin M. H. Bychowski M. E. Auger C. J. Auger A. P. (2010). The nuclear receptor corepressor has organizational effects within the developing amygdala on juvenile social play and anxiety-like behavior. Endocrinology 151 (3), 1212–1220. 10.1210/en.2009-0594 20051490 PMC2840691

[B30] Jones M. J. Goodman S. J. Kobor M. S. (2015). DNA methylation and healthy human aging. Aging Cell 14 (6), 924–932. 10.1111/acel.12349 25913071 PMC4693469

[B31] Kalmar J. H. Wang F. Chepenik L. G. Womer F. Y. Jones M. M. Pittman B. (2009). Relation between amygdala structure and function in adolescents with bipolar disorder. J. Am. Acad. Child. Adolesc. Psychiatry 48 (6), 636–642. 10.1097/CHI.0b013e31819f6fbc 19454919 PMC2867040

[B32] Klose R. J. Bird A. P. (2006). Genomic DNA methylation: the mark and its mediators. Trends Biochem. Sci. 31 (2), 89–97. 10.1016/j.tibs.2005.12.008 16403636

[B33] Kraszpulski M. Dickerson P. A. Salm A. K. (2006). Prenatal stress affects the developmental trajectory of the rat amygdala. Stress 9 (2), 85–95. 10.1080/10253890600798109 16895832

[B34] Kundakovic M. Gudsnuk K. Herbstman J. B. Tang D. Perera F. P. Champagne F. A. (2015). DNA methylation of BDNF as a biomarker of early-life adversity. Proc. Natl. Acad. Sci. U. S. A. 112 (22), 6807–6813. 10.1073/pnas.1408355111 25385582 PMC4460453

[B35] Kundakovic M. Jaric I. (2017). The Epigenetic link between prenatal adverse environments and neurodevelopmental disorders. Genes 8 (3), 104. 10.3390/genes8030104 PMC5368708 28335457

[B36] Lawrie S. M. Whalley H. C. Job D. E. Johnstone E. C. (2003). Structural and functional abnormalities of the amygdala in Schizophrenia. Ann. N. Y. Acad. Sci. 985 (1), 445–460. 10.1111/j.1749-6632.2003.tb07099.x 12724176

[B37] Lay D. C. Jr. Randel R. D. Friend T. H. Jenkins O. C. Neuendorff D. A. Bushong D. M. (1997). Effects of prenatal stress on suckling calves. J. Anim. Sci. 75 (12), 3143–3151. 10.2527/1997.75123143x 9419987

[B38] LeDoux J. E. (1994). The amygdala: contributions to fear and stress. Seminars Neurosci. 6 (4), 231–237. 10.1006/smns.1994.1030

[B39] Littlejohn B. P. Price D. M. Banta J. P. Lewis A. W. Neuendorff D. A. Carroll J. A. (2016). Prenatal transportation stress alters temperament and serum cortisol concentrations in suckling Brahman calves. J. Anim. Sci. 94 (2), 602–609. 10.2527/jas.2015-9635 27065130

[B40] Littlejohn B. P. Price D. M. Neuendorff D. A. Carroll J. A. Vann R. C. Riggs P. K. (2018). Prenatal transportation stress alters genome-wide DNA methylation in suckling Brahman bull calves. J. Anim. Sci. 96 (12), 5075–5099. 10.1093/jas/sky350 30165450 PMC6276578

[B41] Livernois A. M. Mallard B. A. Cartwright S. L. Cánovas A. (2021). Heat stress and immune response phenotype affect DNA methylation in blood mononuclear cells from Holstein dairy cows. Sci. Rep. 11 (1), 11371. 10.1038/s41598-021-89951-5 34059695 PMC8166884

[B42] Mohrhauser D. A. Taylor A. R. Gonda M. G. Underwood K. R. Pritchard R. H. Wertz-Lutz A. E. (2015). The influence of maternal energy status during mid-gestation on beef offspring tenderness, muscle characteristics, and gene expression. Meat Sci. 110, 201–211. 10.1016/j.meatsci.2015.07.017 26253836

[B43] Mychasiuk R. Ilnytskyy S. Kovalchuk O. Kolb B. Gibb R. (2011). Intensity matters: brain, behaviour and the epigenome of prenatally stressed rats. Neuroscience 180, 105–110. 10.1016/j.neuroscience.2011.02.026 21335068

[B44] Papin C. Le Gras S. Ibrahim A. Salem H. Karimi M. M. Stoll I. (2021). CpG Islands shape the epigenome landscape. J. Mol. Biol. 433 (6), 166659. 10.1016/j.jmb.2020.09.018 33010306

[B45] Park H.-J. Kim S.-K. Kang W.-S. Chung J.-H. Kim J.-W. (2014). Increased activation of synapsin 1 and mitogen-activated protein kinases/extracellular signal-regulated kinase in the amygdala of maternal separation rats. CNS Neurosci. Ther. 20 (2), 172–181. 10.1111/cns.12202 24279756 PMC6493014

[B46] Petit B. Boissy A. Zanella A. Chaillou E. Andanson S. Bes Lévy F. (2015). Stress during pregnancy alters dendritic spine density and gene expression in the brain of new-born lambs. Behav. Brain Res. 291, 155–163. 10.1016/j.bbr.2015.05.025 26005125

[B47] Price D. M. Lewis A. W. Neuendorff D. A. Carroll J. A. Burdick Sanchez N. C. Vann R. C. (2015). Physiological and metabolic responses of gestating Brahman cows to repeated transportation. J. Anim. Sci. 93 (2), 737–745. 10.2527/jas.2013-7508 26020755

[B48] Rasia-Filho A. A. Londero R. G. Achaval M. (2000). Functional activities of the amygdala: an overview. J. Psychiatry Neurosci. 25 (1), 14–23. 10721680 PMC1407702

[B49] Razin A. Riggs A. D. (1980). DNA methylation and gene function. Science 210 (4470), 604–610. 10.1126/science.6254144 6254144

[B50] Redlich R. Schneider I. Kerkenberg N. Opel N. Bauhaus J. Enneking V. (2020). The role of BDNF methylation and Val(66) Met in amygdala reactivity during emotion processing. Hum. Brain Mapp. 41 (3), 594–604. 10.1002/hbm.24825 31617281 PMC7268057

[B51] Robinson M. D. McCarthy D. J. Smyth G. K. (2010). edgeR: a Bioconductor package for differential expression analysis of digital gene expression data. Bioinformatics 26 (1), 139–140. 10.1093/bioinformatics/btp616 19910308 PMC2796818

[B52] Rosen B. D. Bickhart D. M. Schnabel R. D. Koren S. Elsik C. G. Tseng E. (2020). De novo assembly of the cattle reference genome with single-molecule sequencing. Gigascience 9 (3), giaa021. 10.1093/gigascience/giaa021 32191811 PMC7081964

[B53] Rosenkranz J. A. Venheim E. R. Padival M. (2010). Chronic stress causes amygdala hyperexcitability in rodents. Biol. Psychiatry 67 (12), 1128–1136. 10.1016/j.biopsych.2010.02.008 20378100 PMC2882519

[B54] Sagalajev B. Wei H. Chen Z. Albayrak I. Koivisto A. Pertovaara A. (2018). Oxidative stress in the amygdala contributes to neuropathic pain. Neuroscience 387, 92–103. 10.1016/j.neuroscience.2017.12.009 29274353

[B55] Salpea P. Russanova V. R. Hirai T. H. Sourlingas T. G. Sekeri-Pataryas K. E. Romero R. (2012). Postnatal development- and age-related changes in DNA-methylation patterns in the human genome. Nucleic Acids Res. 40 (14), 6477–6494. 10.1093/nar/gks312 22495928 PMC3413121

[B56] Sanglard L. P. Nascimento M. Moriel P. Sommer J. Ashwell M. Poore M. H. (2018). Impact of energy restriction during late gestation on the muscle and blood transcriptome of beef calves after preconditioning. BMC Genomics 19 (1), 702. 10.1186/s12864-018-5089-8 30253751 PMC6156876

[B57] Scheinost D. Kwon S. H. Lacadie C. Sze G. Sinha R. Constable R. T. (2016). Prenatal stress alters amygdala functional connectivity in preterm neonates. Neuroimage. Clin. 12, 381–388. 10.1016/j.nicl.2016.08.010 27622134 PMC5009231

[B58] Serviento A. M. Lebret B. Renaudeau D. (2020). Chronic prenatal heat stress alters growth, carcass composition, and physiological response of growing pigs subjected to postnatal heat stress. J. Anim. Sci. 98 (5), skaa161. 10.1093/jas/skaa161 32415838 PMC7245536

[B59] Tate P. H. Bird A. P. (1993). Effects of DNA methylation on DNA-binding proteins and gene expression. Curr. Opin. Genet. Dev. 3 (2), 226–231. 10.1016/0959-437x(93)90027-m 8504247

[B60] Thomas P. D. Campbell M. J. Kejariwal A. Mi H. Karlak B. Daverman R. (2003). PANTHER: A library of protein families and subfamilies indexed by function. Genome Res. 13 (9), 2129–2141. 10.1101/gr.772403 12952881 PMC403709

[B61] Thompson R. P. Nilsson E. Skinner M. K. (2020). Environmental epigenetics and epigenetic inheritance in domestic farm animals. Anim. Reprod. Sci. 220, 106316. 10.1016/j.anireprosci.2020.106316 32094003

[B62] Vonderwalde I. (2019). DNA Methylation within the amygdala early in life increases susceptibility for depression and anxiety disorders. J. Neurosci. 39 (45), 8828–8830. 10.1523/JNEUROSCI.0845-19.2019 31694977 PMC6832684

[B63] Ward H. E. Johnson E. A. Salm A. K. Birkle D. L. (2000). Effects of prenatal stress on defensive withdrawal behavior and corticotropin releasing factor systems in rat brain. Physiol. Behav. 70 (3-4), 359–366. 10.1016/s0031-9384(00)00270-5 11006435

[B64] Wikenius E. Moe V. Smith L. Heiervang E. R. Berglund A. (2019). DNA methylation changes in infants between 6 and 52 weeks. Sci. Rep. 9 (1), 17587. 10.1038/s41598-019-54355-z 31772264 PMC6879561

[B65] Wilson V. L. Smith R. A. Ma S. Cutler R. G. (1987). Genomic 5-methyldeoxycytidine decreases with age. J. Biol. Chem. 262 (21), 9948–9951. 10.1016/s0021-9258(18)61057-9 3611071

[B66] Yang Y. Wang J. Z. (2017). From structure to behavior in basolateral amygdala-hippocampus circuits. Front. Neural Circuits 11, 86. 10.3389/fncir.2017.00086 29163066 PMC5671506

[B67] Ziller M. J. Hansen K. D. Meissner A. Aryee M. J. (2015). Coverage recommendations for methylation analysis by whole-genome bisulfite sequencing. Nat. Methods 12 (3), 230–232. 10.1038/nmeth.3152 25362363 PMC4344394

